# Correlation of Point of Care Ultrasound (POCUS) Guided Pupillary Assessment Parameter with Glasgow Coma Scale in Patients with Altered Mental Status– A Cross Sectional Study 

**DOI:** 10.24908/pocus.v9i2.17449

**Published:** 2024-11-15

**Authors:** Priyanka Modi, Sanjeev Bhoi

**Affiliations:** 1 Department of Emergency Medicine, All India Institute Of Medical Sciences (AIIMS) New Delhi IND

**Keywords:** POCUS, GCS, Pupillometry, Pupillary Light Reflex, Altered Mental Status

## Abstract

**Background:** Earlier studies have proved deteriorating Glasgow Coma Scale (GCS) as a marker of raised intracranial pressure (ICP). Low GCS is associated with abnormal pupillary parameters. Currently, many studies have proved that ultrasound provides a feasible and objective assessment of pupillary light reflex. However, literature is lacking to evaluate objective pupillary parameters to predict GCS of the patients by point of care ultrasound (POCUS). **Materials and methods:** In this prospective, cross-sectional study, 200 patients were recruited in the emergency department. The inclusion criteria were patients older than 18 years with acute presentation of altered mental status. Exclusion criteria were patients who had partial globe rupture or dementia. The patients underwent a B-mode POCUS-guided evaluation at rest and after light stimulation. Statistical analysis of relationship between pupillary assessment parameters and GCS was performed using Spearman's Rank correlation coefficient, Kruskal-Wallis equality-of-populations rank test, and area under the receiver operating characteristic.** Results: **The study consisted of 200 (42 female, 158 male) patients with mean (± standard deviation) of age and GCS of 43.56 ± 16.50 years and 5.54 ± 3.00, respectively. Majority of non-reactive pupils had a GCS Score of 3-8 (74 cases, 97.37%). The Pupillary diameter (PD) and PD variation showed statistically significant agreement with pupil reactivity to light stimulation and GCS, with Spearman's correlation coefficient ranging from 0.28 to 0.33 for PD, and -0.55 to -0.50 (p-value <0.05) for PD variation, respectively. PD variation rate (PDVR) is the percentage change in the magnitude of constriction of PD on light stimulation. PDVR of >19.68% had a sensitivity of 86.96% (95% CI: 82.04 - 91.88%) and specificity of 64.97% (95% CI: 58.00 - 71.94%) to detect GCS>8. **Conclusion:** PD variation and PDVR measured by POCUS has significant correlation with GCS >8. The study showed good sensitivity and low specificity of PDVR on light stimulation to detect GCS >8.

## Introduction

The emergency department has a major role in the management of acute presentation of altered sensorium 1. Altered sensorium may be associated with a wide range of conditions. Standardized evaluation is essential to measure their clinical deterioration or improvement, as well as to predict outcome and response to treatment and to guide further management. Currently, the Glasgow Coma Scale (GCS) and intracranial pressure (ICP) measurement are used for monitoring them. The GCS was originally introduced as a tool to assess levels of consciousness in patients with acute head trauma, and now, is widely used in all patients with altered sensorium 2. However, using invasive ICP measurement devices is not feasible in the emergency department. Hence, non-invasive methods like pupillometry have been proposed. The increase in ICP is associated with abnormal pupilometer readings 3, 4, 5, 6. Pupilometer reading, founded and described first by Lowestein in his writings in 1958, carefully analyzes abnormal pupillary assessment parameters like Pupillary diameter (PD) and shape which helps in definite clinical diagnosis based on the existence and location of lesions within the nervous network of pupillary control 7. In addition, video assessment or infrared pupillometry devices are being used to monitor raised ICP in critically ill patients and have changed the accuracy and reliability of the pupillary light reflex (PLR) assessment. In particular, its ability to measure pupillary function in a fast, non-invasive, reliable, and quantifiable manner is of great help in the clinical diagnosis of neuro-critically ill patients in intensive care units and emergency departments 8. However, these instruments are rarely available in emergency departments. In contrast, B-mode point of care ultrasound (POCUS) is a simple, innovative, widely available, non-invasive, time- and cost-effective imaging technique to document routine pupillary light reflex examination. Currently, many studies have proved feasibility of pupillometry by POCUS 9, 10. A previous study by Yic et al. established strong correlation between POCUS pupillary assessment with infrared pupillary assessment in critically ill patients 9. However, literatures are lacking in describing the relationship between POCUS pupillometry assessment parameters and GCS. The objective of this study was to establish a relationship between the parameter of PLR assessment by POCUS and GCS.

## Materials and methods

### Study design and participants

**Figure 1:**
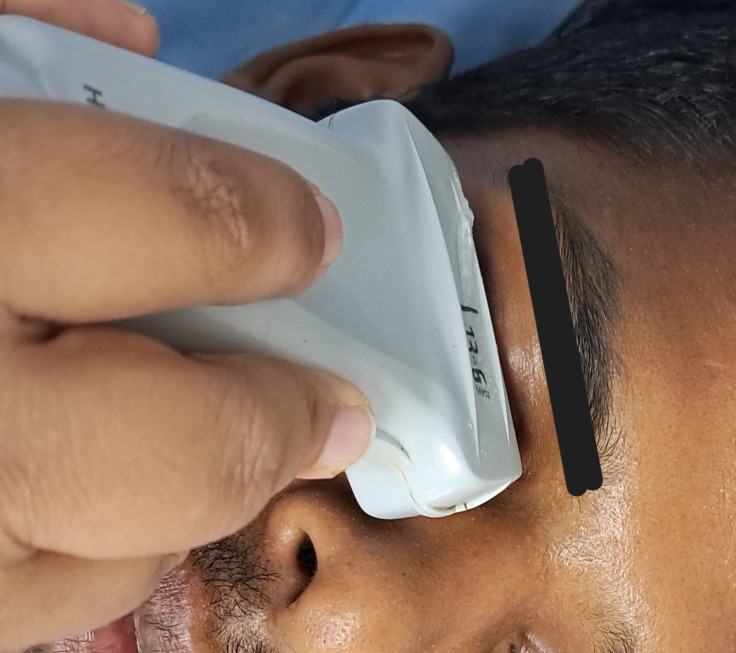
Proper position of the linear probe of POCUS for assessment of pupillary function by B-mode ultrasound. POCUS, point of care ultrasound.

In this prospective, cross-sectional study, study cases were recruited following the Helsinki Declaration and approved by the Institute Ethics Committee, from the Department of Emergency Medicine. The inclusion criteria were patients older than 18 years with acute presentation of altered mental status. Exclusion criteria were patients who did not give consent, less than 18 years old, having partial globe rupture and dementia. After the written consent from a close family member present at the time of examination, 200 patients were evaluated by B-mode POCUS-guided study at rest and after light stimulation using linear probe and saved (Figure 1). The duration of POCUS examination was never more than 5 minutes. The study was conducted from January 1,2019, to February 28,2021, by a resident with six month’s experience in POCUS. The resident was taught about body ergonomics, image acquisition, optimization and interpretation. 

### POCUS pupillometry process during pupillary light reflex examination

**Figure 2:**
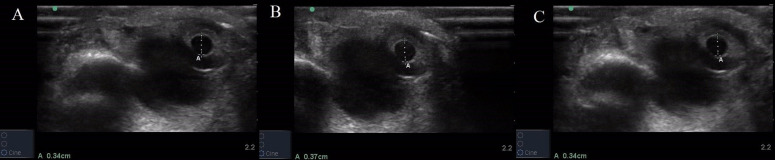
PD assessment in closed eye by B-mode ultrasound. Crosses represent the markers set by the examiner for measuring PD. A, PD at rest. B, PD on direct light stimulation. C, PD on consensual light stimulation. PD, pupillary diameter.

The POCUS scanning process of pupil during PLR examination included evaluation of bilateral PD and PLR. All insonations were performed by the same resident doctor in supine position under room lighting with the subject's eyes closed. B-mode POCUS Scanning process was selected with minimum power settings according to the ALARA (as low as reasonably achievable) insonation approach (Figure 1). The details of patients were filled in the predesigned form. Completely closed eyelids were wiped with normal saline. The linear array probe (6 – 13 MHz) was gently positioned flatly over the lower edge of the closed eye for a trans-palpebral tangential view, with the probe marker pointed toward the right side of the patient, with appropriate depth setting so that the image acquisition, image optimization & interpretation could be done with precision. Torchlight was used over the closed eyes for PLR examination (Figure 2).

**Figure 3:**
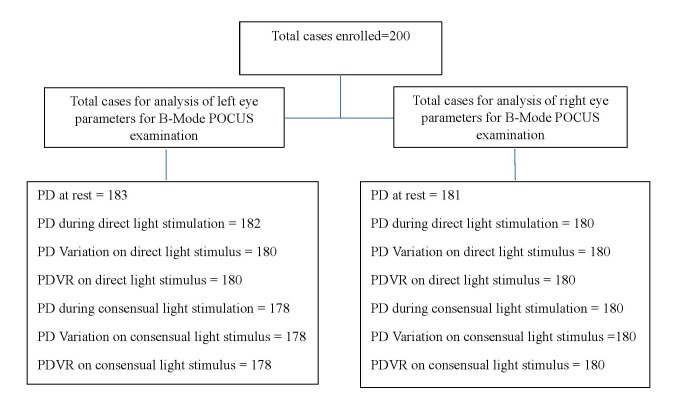
Flow chart of the included patients for B-mode POCUS examination of different pupillary assessment parameters on light stimulation. POCUS, point of care ultrasound. PD, pupillary diameter.

### Statistical analysis

Figure 3 shows flow diagram of the included patients for B-mode POCUS examination of different pupillary assessment parameters on light stimulation. The collected data were filled in Microsoft Excel followed by analysis using SPSS software (IBM SPSS Statistics 26.0). Kolmogorov-Smirnov and Shapiro-Wilk tests were applied to check the normality of continuous variable data. The nominal variables were expressed as frequencies with percentage and continuous variables as mean ± standard deviation and median (Interquartile range, minimum, maximum). The various ultrasonographic pupillometry assessment parameters were PD, PD variation, and PD variation rate (PDVR). The PD variation is the magnitude of constriction of PD after either direct or consensual light stimulation and was calculated using the equation: (PD under natural light – PD under light reflex). The PDVR is the percentage change in the magnitude of constriction of PD on light stimulation and was determined using the equation: [(PD under natural light – PD under light reflex) X 100] /PD under natural light. 

Spearman's Rank correlation coefficient was used to show how pupillary assessment parameters change with pupil reactivity to light stimulation and with GCS status. The Spearman Rank Correlation consists of values from +1 to -1, indicating strength of association as follows: a value of +1 perfect association, ≥0.8 strong positive association, 0.4 to 0.8 moderate positive association, 0 to 0.4 weak positive association, 0 no association, 0 to -0.4 weak negative association, -0.4 to -0.8 moderate negative association, -0.8 to -1 strong negative association, and a value of -1 perfect negative association of rank). The null hypothesis (confidence level of 95% with a p-value of 0.05) was that there was no correlation between them. A p-value ≤0.05 was considered statistically significant 11. 

Kruskal-Wallis equality-of-populations rank test was used to analyze the relationship between POCUS pupillometry assessment parameters within the three GCS groups. 

The sensitivity of PDVRs on light stimulation to detect pupil reactivity and to detect better GCS status (GCS Score>8) was evaluated by analyzing the PDVRs based on the area under the receiver operating characteristic (AUROC) curve. Overall model quality was used to rule out random prediction of receiver operating characteristic (ROC) for all variables (<0.5 means the model is no better than random prediction).

## Results

### Sample characteristics

Two hundred (42 female, 158 male) patients with mean (± standard deviation) of age and GCS of 43.56 ± 16.50 years and 54 ± 3.00, respectively, were recruited into the study. They were further divided for group analysis into three GCS groups based on severity of GCS just like head injury [group 1 (G1), 3-8 scores; group 2 (G2), 9-12 scores; group 3 (G3), 13-14 scores]. The grouping helped to determine the cutoff for pupillary parameter variables in cases with severely low GCS Scores (<8). Out of 200 cases for POCUS examination of PLR, 111 (55.5%) were reactive [Unilateral (U/L) or Bilateral (B/L)], 76 (38%) were non-reactive and 13 (6.5%) had unsuccessful B/L POCUS examination. Majority of non-reactive pupils had GCS Score of 3-8 (74 cases, 97.37%) in comparison to GCS Score of 9-14 (2 cases, 2.63%). However, the reactive group had 87 cases (78.38%) with GCS Score of 3-8, and 24 cases (19.62%) with GCS Score of 9-14. The causes of these unsuccessful USG examinations of PLR (27 cases) were non-cooperative patients (13), phthisis bulbi (4), shrunken eyelids (2), globe rupture (2), periorbital oedema (3), bulged eyeball (2), and artificial eyeball (1). 

**Table 1:** Patient pupillary light reflex assessment results with respect to GCS Score and pupil reactivity to light stimulation on Spearman Correlation

Variables	Number of Cases (N)	Mean ± Std. Deviation (mm) Minimum, Maximum (mm)	Median (mm) IQR (mm)	Spearman Correlation Coefficient (PD with GCS)	P-Value (PD with GCS)	Spearman correlation coefficient (PD with pupillary reactivity)	P-value (PD with pupillary reactivity)
**PD at rest **							
Left eye	183	2.95 ± 1·17 (1.10, 6.10)	2.90 (1.90-3.80)	-0.23	0.002	0.14	0.06
Right eye	181	3.03 ± 1·24 (1.00, 6.40)	3.00 (1.85-4.00)	-0.24	0.001	0.10	0.20
**PD during direct light stimulation **							
Left eye	182	2.50 ± 1·18 (0.80, 6.10)	2.15 (1.60- 3.20)	-0.38	<0.001	0.32	<0.001
Right eye	180	2.57 ± 0·57 (0.70, 6.45)	2.25 (1.60- 3.40)	-0.33	<0.001	0.28	<0.001
**PD variation on direct light stimulus **							
Left eye	180	0.46 ± (-0.40, 3.00)	0.30 (0-0.70)	0.36	<0.001	-0.51	<0.001
Right eye	180	0.46 ± (-0.90, 2.90)	0.30 (0.10-.79)	0.27	<0.001	-0.55	<0.001
**PD during consensual light stimulation **							
Left eye	178	2.46 ± 1·22 (0.90, 6.40)	2.10 (1.50-3.30)	-0.38	<0.001	0.33	<0.001
Right eye	180	2.46 ± 1·24 (0.70, 6.30)	2.15 (1.50-3.20)	-0.32	<0.001	0.30	<0.001
**PD Variation on consensual light stimulus **							
Left eye	178	0.50 ± (-0.30, 2.50)	0.30 (0.04-0.90)	0.30	<0.001	-0.50	<0.001
Right eye	180	0.57 ± (-0.60, 3.00)	0.40 (0.10-1.00)	0.17	0.022	-0.52	<0.001

IQR, Interquartile range. PD, Pupillary Diameter

**Figure 4:**
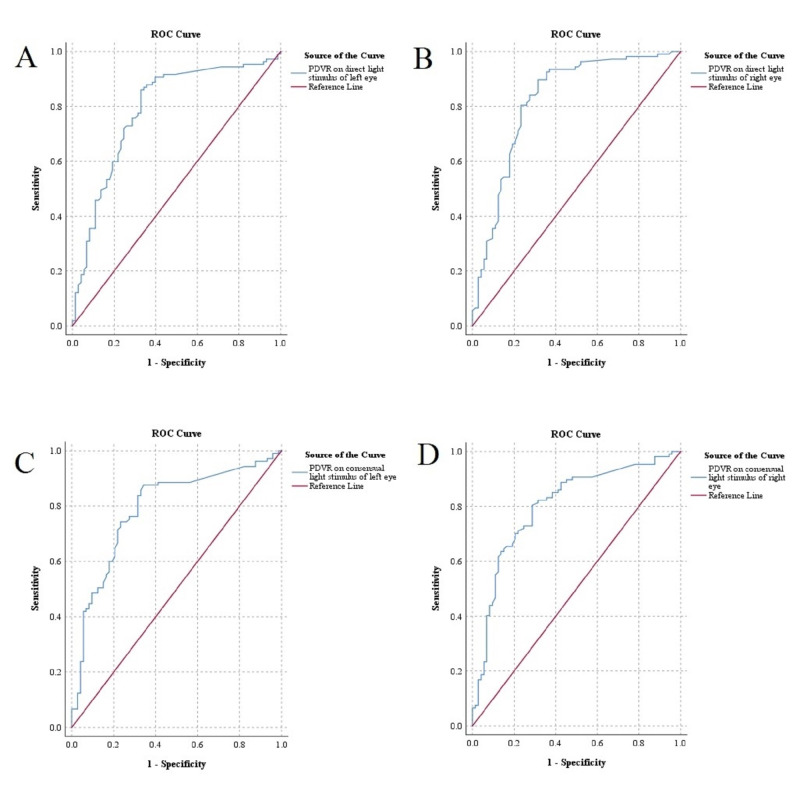
ROC curve of PD variation based on better pupil reactivity (reactive or non-reactive). Diagonal segments are produced by ties. A, PDVR on direct light stimulus of left eye by pupil reactivity. B, PDVR on direct light stimulus of right eye by pupil reactivity. C, PDVR on consensual light stimulus of left eye by pupil reactivity. D, PDVR on consensual light stimulus of right eye by pupil reactivity. ROC, Receiver operating characteristic. PD, Pupillary diameter. PDVR, Pupillary diameter variation rate.

**Table 2:** Patient pupillary light reflex assessment results with respect to GCS Score groups on Kruskal-Wallis equality-of-populations rank test.

Variables	Number of Cases (N)	P-value	G1 GCS Score 3-8 (N=169) Mean ± Std. Deviation	G2 GCS Score 9-12 (N=24) Mean ± Std. Deviation	G3 GCS Score 13-14 (N=7) Mean ± Std. Deviation
**PD at rest **					
Left eye	183	G1-G2-G3 (0.72)	2.94 ± 1.19 2.90 (1.90, 3.80)	2.89 ± 1.05 2.80 (2.00, 3.80)	3.32 ± 1.21 2.80 (2.30, 4.60)
Right eye	181	G1-G2-G3 (0.93)	3.04 ± 1.28 3.00 (1.80, 4.10)	2.92 ± 1.00 3.00 (2.20, 3.70)	3.08 ± 0.68 3.20 (2.45, 3.65)
**PD variation on direct light stimulus **					
Left eye	182	G1-G2-G3 (0.12)	2.58 ± 1.22 2.20 (1.60, 3.45)	1.91 ± 0.55 1.80 (1.60, 2.20)	2.43 ± 0.84 2.45 (1.65, 3.18)
Right eye	180	G1-G2-G3 (0.39)	2.64 ± 1.32 2.30 (1.60, 3.70)	2.07 ± 0.57 2.10 (1.58, 2.35)	2.10 ± 0.63 1.90 (1.60, 2.70)
**PD variation on direct light stimulus **					
Left eye	180	G1-G2-G3 (<0.001); G1-G2 (<0.001) G1-G3 (0.015) G2-G3 (0.677)	0.38 ± 0.49 0.30 (0, 0.60)	0.98 ± 0.67 0.90 (0.40, 1.40)	1.08 ± 0.50 1.20 (0.55, 1.48)
Right eye	180	G1-G2-G3 (0.001); G1-G2 (0.002) G1-G3 (0.014) G2-G3 (0.494)	0.39 ± 0.54 0.30 (0.10, 0.60)	0.89 ± 0.75 0.75 (0.30, 1.23)	0.98 ± 0.47 1.00 (0.60, 1.35)
**PD during consensual light stimulation**					
Left eye	178	G1-G2-G3 (0.18)	2.53 ± 1.25 2.10 (1.50, 3.40)	2.00 ± 0.76 1.70 (1.50, 2.60)	1.87 ± 0.98 1.30 (1.30, 1.30)
Right eye	180	G1-G2-G3 (0.36)	2.54 ± 1.30 2.20 (1.50, 3.40)	1.91 ± 0.42 1.90 (1.64, 2.20)	2.02 ± 0.49 2.10 (1.55, 2.45)
**PD variation on consensual light stimulus **					
Left eye	178	0.50 ± (-0.30, 2.50)	0.30 (0.04-0.90)	0.30	<0.001
Right eye	180	0.57 ± (-0.60, 3.00)	0.40 (0.10-1.00)	0.17	0.022

PD, pupillary diameter. G1, group 1; GCS Score 3-8, group 2; GCS Score 9-12; G3, group 3. GCS score 13-14; IQR, Interquartile Range

**Figure 5:**
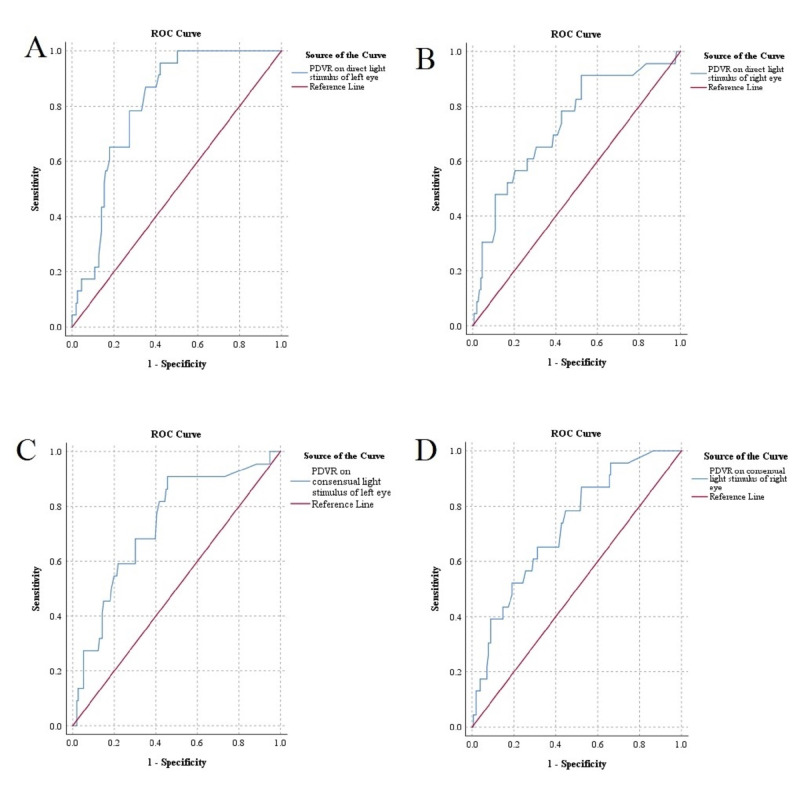
ROC curve of PDVR based on better GCS status (GCS >8). Diagonal segments are produced by ties. A, PDVR on direct light stimulus of left eye by GCS status. B, PDVR on direct light stimulus of right eye by GCS status. C, PDVR on consensual light stimulus of left eye by GCS status. D, PDVR on consensual light stimulus of right eye by GCS status. ROC, Receiver operating characteristic. PD, Pupillary diameter. PDVR, Pupillary diameter variation rate.

**Table 3:** Patient PDVR during pupillary light reflex assessment with USG results with respect to GCS Score groups

PDVR Variables (N)	Mean±Std Deviation (%) Number of cases (N)	Median (mm) Interquartile range (mm)	P-value	G1 GCS Score (3-8)(N=169)		G2 GCS Score (9-12) (N=24)		G3 GCS Score (13-14) (N=7)	
				Median (IQR) (%)	Mean±Std. Deviation (Minimum. Maximum)(%)	Median (IQR) (%)	Mean±Std. Deviation (Minimum. Maximum) (%)	Median (IQR) (%)	Mean±Std. Deviation (Minimum. Maximum) (%)
PDVR on direct light stimulus									
Left eye 180	15.75 ± 16.55	14.29 (0- 29.41)	G1-G2-G(<0.001) G1-G2 (<0.001) G1-G3 (0.041) G3-G2 (0.978)	11.36 (0,26.58)	13.53±16.11 (-21.43,56.82)	32.0 (24.39,34.88)	31.15 ± 11.45 (10.71, 57.69)	31.51 (22.78,34.76)	29.68 ± 6.76 (20.00,35.71)
Right eye 180	15.47 ± 16.31	15.00 (2.90- 26.53)	G1-G2-G3(0.001) G1-G2 (0.003) G1-G3 (0.018) G3-G2 (0.494)	13.33(2.33,24.07)	13.73 ± 15.53 (-27.12, 60.47)	27.29 (15.16, 42.35)	26.19 ± 17.81 (-11.76, 59.18)	30.30 (18.07,45.37)	31.44 ± 14.40 (13.64, 50.00)
PDVR on consensual light stimulus									
Left eye 180	15.75 ± 16.55	14.29 (0- 29.41)	G1-G2-G(<0.001) G1-G2 (<0.001) G1-G3 (0.041) G3-G2 (0.978)	11.36 (0, 26.58)	13.53 ± 16.11 (-21.43, 56.82)	32.0 (24.39, 34.88)	31.15 ± 11.45 (10.71, 57.69)	31.51 (22.78,34.76)	29.68 ± 6.76 (20.00, 35.71)
Right eye 180	15.47 ± 16.31	15.00 (2.90- 26.53)	G1-G2-G3 (0.001) G1-G2 (0.003) G1-G3 (0.018) G3-G2 (0.494)	13.33 (2.33,24.07)	13.73 ± 15.53 (-27.12, 60.47)	27.29 (15.16, 42.35)	26.19 ± 17.81 (-11.76, 59.18)	30.30 (18.07,45.37)	31.44 ± 14.40 (13.64, 50.00)
PDVR on consensual light stimulus									
Left eye 178	17.42 ± 17.56	16.45 (1.06- 30.47)	G1-G2-G3(0.001) G1-G2 (0.003) G1-G3 (0.016) G3-G2 (0.272)	10.53 (0, 28.92)	15.59 ± 17.00 (-17.65, 56.82)	30.0 (18.18, 46.67)	28.77 ± 16.55 (-5.26, 53.13)	35.0 (33.33, 35.0)	40.63 ± 11.23 (33.33, 53.57)
Right eye 180	18.68 ± 17.83	18.20 (2.04- 31.58)	G1-G2-G3 (0.002) G1-G2 (0.005) G1-G3 (0.015) G3-G2 (0.410)	15.00 (0, 30.0)	16.82 ± 17.25 (-15.38, 61.29)	28.24 (13.33, 46.09)	30.48 ± 18.91 (0.00, 61.22)	33.33 (29.89,39.41)	34.38 ± 6.25 (27.27, 44.44)

**Table 4:** Diagnosis of pupillary reflex (reactivity) using PDVR

**Test Result Variable(s)**	**Number of cases**	**AUC**	**P-value**	**95% Confidence Interval (Lower-Upper Limits)**	**Cutoff** **(%)**	**Youden’s index**	**Sensitivity** **(95% CI)** **(%)**	**Specificity** **(95% CI)** **(%)**
**PDVR on direct light stimulus**								
Left eye	180	0.786	<0.001	0.716 - 0.857	6.56	0.53	85.98 (80.91-91.05)	67.12 (60.26-73.99)
Right eye	180	0.823	<0.001	0.756 - 0.889	7.18	0.58	89.72 (85.28-94.16)	68.49 (61.71-75.28)
**PDVR on consensual light stimulus**								
Left eye	178	0.791	<0.001	0.721 - 0.860	7.60	0.52	83.81 (78.40-89.22)	67.12 (60.22-74.02)
Right eye	180	0.802	<0.001	0.735 - 0.869	10.62	0.51	82.24 (76.66-87.83)	68.49 (61.71-75.28)

AUC, Area Under Curve; CI, Confidence Interval

### Correlation between pupillary parameters at rest and during light stimulus with pupillary reactivity and GCS Scores

onsists of the descriptive statistics and results of correlation between pupillary parameters and pupillary reactivity (reactive or non-reactive) on light stimulation as well with GCS Scores. The PD and PD variation showed statistically significant agreement (p-value <0.05) with pupil reactivity to light stimulation, except PD at rest. Spearman’s correlation coefficients between PD and pupillary reflex activity on direct light stimulation of left and right pupil were 0.32 and 0.28, respectively; and on consensual light stimulation of left and right pupil were 0.33 and 0.30, respectively. This showed larger PD has better pupillary reflex activity. In addition, the Spearman’s correlation coefficient between PD variation and pupillary reflex activity on direct light stimulation of left and right pupil were -0.51 and -0.55, respectively; and on consensual light stimulation of left and right pupil were -0.50 and -0.52, respectively. This showed a decrease in PD has better pupillary reflex activity.

Similarly, pupillary parameters showed statistically significant agreement (p-value <0.05) with GCS Score. The Spearman’s correlation coefficient between PD and GCS of left and right pupil at rest were -0.23 and -0.24, respectively; on direct light stimulation were -0.38 and -0.33; on consensual light stimulation of left and right pupil were -0.38 and -0.32, respectively. This showed that larger PD has lower GCS. In addition, Spearman’s correlation coefficients between PD variation and GCS on direct light stimulation of left and right pupil were 0.36 and 0.27, respectively; and on consensual light stimulation of left and right pupil were 0.30 and 0.17, respectively. This showed a decrease in PD on light stimulation had better GCS.

### The distribution of parameters of PD variables across categories of GCS groups

onsists of results of comparison of pupillary parameters across categories of GCS groups. On Kruskal-Wallis equality-of-populations rank test, the distribution of PD at rest and on light stimulation of B/L eyes were the same across categories of GCS groups (p-value ≥ 0.05) and that of PD variation/PDVR on light stimulation of B/L eyes were different (p-value <0.05) (Tables 2 and 3). There was a significant increase in the magnitude of PD variation and PDVR on light stimulation between G1 and G2, and between G1 and G3 (p-value <0.05). The median (IQR) (mm) PD variation ranged from 0.30 mm (0, 0.60) to 0.30 mm (0, 0.80) in G1, and 0.75 mm (0.30, 1.23) to 1.50 mm (0.70, 1.50) in G2 and G3 (Table 2). The median (IQR) (%) of PDVR of B/L eyes on light stimulation (direct/ consensual) ranged from 10.53% (0, 28.92) to 15.00% (0, 30.0) in G1, and 27.29% (15.16, 42.35) to 35.0% (33.33, 35.0) in G2 and G3 (Table 3).

### Diagnostic sensitivity of PDVR based on pupillary reactivity to light stimulation 

Figure 4 and Table 4 show the AUROC and analysis of sensitivity of ultrasonic PDVR based on pupillary reactivity to light stimulation. ROC for PDVRs were tested for overall model quality. The overall model quality of ROC for PDVR on light stimulus were >0.5 (0.72-0.76). For PDVR of left and right eye on direct light stimulus (respectively), the cut-off values of PDVR were 6.56% and 7.18%,; area under curve (AUC) values were 0.79 and 0.82,; sensitivity were 85.98% (95% CI: 80.91-91.05) and 89.72% (95% CI: 85.28-94.16); and specificity were 67.12% (95% CI: 60.26-73.99) and 68.49% (95% CI: 61.71-75.28). For PDVR of left and right eye on consensual light stimulus (respectively), the cut-off values of PDVR were 7.60% and 10.62%; AUC values were 0.79 and 0.80; sensitivity were 83.81% (95% CI: 78.40-89.22) and 82.24% (95% CI: 76.66-87.83); and specificity were 67.12% (95% CI: 60.22-74.02) and 68.49% (95% CI: 61.71-75.28).

### Diagnostic sensitivity of PDVR based on better GCS status (GCS>8) in patients with altered sensorium

Figure 5 and Table 5 show the AUROC and the analysis of sensitivity of ultrasonic PDVR based on better GCS status (GCS>8). GCS Score 3-8 was considered as poor GCS status and was considered less sensitive to produce higher PDVR (%). However, GCS Score >8 was considered a better GCS status and was considered more sensitive to produce higher PDVR (%). The overall model quality of ROC for PDVR on light stimulus (direct or consensual) B/L eyes were >0.5 (0.62-0.73). For PDVR of left and right eye on direct light stimulus (respectively), the cut-off values of PDVR were 19.68% and 11.88%; AUC values were 0.80 and 0.73; sensitivity were 86.96% (95% CI: 82.04-91.88) and 86.96% (95% CI: 82.04- 91.88); and specificity were 64.97% (95% CI: 58.00-71.94) and 47.77% (95% CI: 40.47-55.07). For PDVR of left and right eye on consensual light stimulus (respectively), the cut-off values of PDVR were 17.92% and 12.92%,; AUC values were 0.74 and 0.73; sensitivity were 81.82% (95% CI: 76.15-87.48) and 86.96% (95% CI: 82.04-91.88); and specificity were 58.33% (95% CI: 51.09-65.58) and 47.77% (95% CI: 40.47-55.07). 

**Table 5:** Diagnosis of good GCS Status Group using PDVR

**Test Result Variable(s)**	**Number of cases**	**AUC**	**P-value**	**95% Confidence Interval (Lower-Upper Limits)**	**Cutoff** **PDVR** **(%)**	**Youden’s index**	**Sensitivity** **(95% CI)** **(%)**	**Specificity** **(95% CI)** **(%)**
**PDVR on direct light stimulus**								
Left eye	180	0.804	<0.001	0.732 -0.875	19.68	0.52	86.96 (82.04- 91.88)	64.97 (58.00-71.94)
Right eye	180	0.734	<0.001	0.621 - 0.846	11.88	0.39	86.96 (82.04- 91.88)	47.77 (40.47-55.07)
**PDVR on consensual light stimulus**								
Left eye	178	0.735	<0.001	0.625 - 0.845	17.92	0.40	81.82 (76.15-87.48)	58.33 (51.09- 65.58)
Right eye	180	0.726	<0.001	0.622 - 0.831	12.92	0.39	86.96 (82.04-91.88)	47.77 (40.47- 55.07)

AUC, Area Under Curve; CI, Confidence Interval; PDVR, Pupillary diameter variation rate

## Discussion

B-mode POCUS is an established method to measure pupil size objectively. However, the role of B-mode POCUS of PLR in the assessment of its relationship with GCS has not been systematically studied so far 12. Here, we report on the application of B-mode POCUS-based pupillary assessment as a novel method for predicting better GCS status (>8) among patients presenting to emergency department with altered sensorium. 

Currently, PD can be accurately measured by transorbital ultrasound probe 9, 13, 14. This helps identify functional deterioration in the central nervous system. Previous research has tentatively demonstrated that an abnormal pupillary light reflex on pupillometry can be indicative of a raised ICP. There is growing evidence that pupillometry holds value in raised ICP management and can be used as an adjunctive neuromonitoring modality alongside monitoring of ICP beyond surrogacy 15. For the diagnosis of raised ICP in the emergency department, doctors are currently dependant on clinical signs such as the GCS, pupillary assessment and/or Cushing's reflex (hypertension, bradycardia and an irregular respiration). However, clinical signs (SBP >160 mm Hg, HR <60 bpm and a fixed pupil >5 mm) are poor predictors of raised ICP and Cushing's sign with altered mental status in traumatic Brain Injury (TBI) is a weak but significant predictor of the need for immediate neurosurgical intervention 16, 17. Also, ICP and Central Perfusion Pressure (CPP) have significant agreement with PLR and GCS of the patient 18. However, there is no consensus about which pupillary assessment parameter is sensitive in detecting a deteriorating GCS status due to raised ICP in the emergency department. 

This study consisted of 55.5% reactive (U/L or B/L) and 38% non-reactive pupils. Of these, 97.37% of non-reactive and 78.38% of reactive pupils had GCS Scores of 3-8. This showed that the majority of non-reactive pupils had low GCS Score (3-8). Hence, this predicted the relation of pupil reactivity with GCS. It was observed that the PD and PD variation showed weak to moderate statistically significant agreement with pupil reactivity to light stimulation (p-value<0.001). In addition, all the pupillary assessment parameters showed weak but statistically significant agreement with GCS Score (p-value <0.01). We wanted to know which GCS group had strong agreement with pupillary assessment parameters. To assess this, we divided GCS status of patients into three groups [G1 3-8 scores, G2 9-12 scores and G3 13-14 scores] to study the distribution of PD variables across categories of GCS groups.

Previous studies have proved no correlation between PD and ICP, and significant correlation of GCS with ICP. They proposed possible quantification of PD variation on light stimulation through the development of proper index 13, 18. Low GCS is a predictor of raised ICP. Hence, our study tried to find which pupillary assessment parameter may change with GCS status. This study showed no agreement between PD at rest or on light stimulation with GCS groups (p-value ≥0.05). However, PD variation and PDVR were different across categories of GCS groups (p-value <0.05). This study showed that the variation in PD on light stimulation predicts a change in GCS status. The median (mm) (IQR) PD variation on light stimulation was larger for GCS 9-14 than that of GCS ≤8.

Similarly, the median (%) (IQR) of PDVR of B/L eyes on light stimulation was larger for GCS 9-14 [27.29% (15.16, 42.35) to 35.0% (33.33, 35.0)] than that of GCS ≥8 [10.53% (0, 28.92) to 15.00% (0, 30.0)]. Hence, there was a significant increase in the magnitude of PD variation and PDVR of B/L eyes on light stimulation from low GCS (≤8) to high GCS (>8) groups (p-value <0.05). However, there was no significant increase in the magnitude of PD variation and PDVR of B/L eyes on light stimulation from moderate GCS (9-12) to high GCS group (13-14).

We found PDVR has excellent ability to diagnose pupil reactivity in patients with altered sensorium. The study showed cutoff values of PDVR to detect pupil reactivity were between 6.56-10.62%; sensitivity to detect pupil reactivity was good and between 82.24-89.72%; specificity to detect pupil reactivity was low and between 67.12-68.49%. Hence, PDVR of >10.6% has sensitivity of >80% to detect reactive pupils. However, PDVR has low specificity (<68.5%) to detect reactive pupils. This can be further studied to develop an objective parameter to predict raised ICP and impending brain herniation. Previous studies have already proved association between automated evaluation of the PLR measurements and raised ICP values 4, 19, 20. 

Motor scores are dynamic and generally deteriorate with time. However, pupillary reactivity is more stable over time. A study by Thakur B et al. proved that a higher GCS is associated with faster dilation velocity of pupils 6, 21. However, our study was based on B-mode POCUS guided pupillometry. While we could study variation in pupil size, we were not able to calculate pupillary constriction time (PCT) and pupillary dilation time (DCT) to analyze constriction and dilation velocity of pupil, respectively. During our study, we observed that the higher PDVR (%) on light stimulation predicted higher GCS status (>8). The AUC values of PDVR based on better GCS status were considered acceptable (0.73-0.80). The study showed cutoff values of PDVR to detect GCS>8 was between 11.88-19.68%, sensitivity to detect GCS>8 was good (81.82-86.96%); specificity to detect GCS>8 was low (47.77-64.97%). Hence, this study concluded that PDVR of >19.7% has sensitivity of >80% to detect GCS>8. However, PDVR is specifically low (<65%) to detect GCS>8. 

## Limitation

B-mode POCUS was used to study pupillary size variation on light stimulation. Since M-mode is used to calculate PCT and DCT, this current study was not able to study either PCT or DCT. As well, this study used normally distributed data. As a result, mean values of PDVR on light stimulation for different GCS groups were not precise and accurate. Future studies may include M-mode along with B-mode ultrasound for PLR examination with a larger population size for more precise and accurate mean values of PDVR.

## Conclusion

PD variation and PDVR measured by POCUS has significant correlation with GCS (>8). The study showed good sensitivity (>80%) and low specificity (<65%) of PDVR (>19.7%) on light stimulation to detect GCS >8. Additionally, PDVR of >10.6% has good sensitivity (>80%) and low specificity (<68.5%) to detect reactive pupils. Ultrasound proved to be a good tool to objectively assess pupillary assessment parameters in patients and their relationship with GCS (non-invasive method to monitor raised ICP) in emergency department and intensive care units. 

## Statement of ethics approval/consent

The study was approved by the Institute Ethics Committee (IECPG-39/23.01.2019, RT-11/28.02.2019)

## Disclosures

The authors do not report any conflict of interest
